# The effect of perceived social support during early pregnancy on depressive symptoms at 6 weeks postpartum: a prospective study

**DOI:** 10.1186/s12888-019-2188-2

**Published:** 2019-07-29

**Authors:** Yuexin Gan, Ran Xiong, Junjiao Song, Xinli Xiong, Fei Yu, Weiming Gao, Hui Hu, Jinsong Zhang, Ying Tian, Xiaobo Gu, Jun Zhang, Dan Chen

**Affiliations:** 1 Ministry of Education-Shanghai Key Laboratory of Children’s Environmental Health, Xinhua Hospital, Shanghai Jiao Tong University School of Medicine, 1665 Kongjiang Road, Shanghai, 200092 China; 2 Department of Neurology, East Hospital, Tongji University School of Medicine, Shanghai, 200120 China; 3 Department of Medical Psychology, Xinhua hospital affiliated to Shanghai Jiao Tong University School of Medicine, Shanghai, China

**Keywords:** Postpartum depression, Postpartum depressive symptoms, Social support, Pregnancy, Prospective study

## Abstract

**Background:**

Postpartum depression was associated with maternal suffering and diminished functioning, increased risk of marital conflict as well as adverse child outcomes. Perceived social support during pregnancy was associated with postpartum depression among women. However, its causal relationship remains unclear. Therefore, we prospectively evaluate the association between perceived social support during early pregnancy and postpartum depressive symptoms.

**Methods:**

We prospectively examined whether perceived social support during early pregnancy affected depressive symptoms at 6 weeks postpartum in a cohort of 3310 women. Perceived social support and postpartum depression were assessed by ENRICHD Social Support Instrument (ESSI) and the postpartum Edinburgh Postpartum Depression Scale (EPDS), respectively. Prevalence of postpartum depressive symptoms was 11.4% (EPDS cutoff≥10). As a test of heterogeneity of association in subpopulations, logistic regression models were performed to analyze the association between social support and postpartum depressive symptoms in strata which were defined by the potential confounder candidates. After multiple imputation, multivariable logistic regression was performed to assess the effect of social support on postpartum symptoms in individual items and total score. Two models were built. Model I adjusted for the variables associated with social support or postpartum depression and changed the association estimates by ≥10%. Model II adjusted for all variables that may be related to social support or postpartum depression.

**Results:**

Significant associations between low perceived social support and postpartum depressive symptoms was found(Model I odds ratio: 1.63, 95% confidence interval: 1.15, 2.30; Model II odds ratio: 1.77, 95% confidence interval: 1.24–2.52). Stratified analyses showed that there was little evidence of heterogeneity of association in subpopulations by basic characteristics of participants.

**Conclusions:**

These findings suggest that early intervention may be able to help protect against depression symptoms at 6 weeks postpartum.

**Electronic supplementary material:**

The online version of this article (10.1186/s12888-019-2188-2) contains supplementary material, which is available to authorized users.

## Background

Postpartum depression (PPD), one of the most common complications of childbearing, affects 5.5–25% of women within 4–8 weeks of giving birth in China [1–5]. It is related to maternal suffering and diminished functioning [6], increased risks of marital conflict in addition to adverse child outcomes including impaired behavioral, emotional, cognitive development and physical health in the children [7, 8]. Maternal suicide and infanticide may be extreme outcomes of PPD [9, 10]. Although it may naturally recover, women with postpartum depression may still suffer from depression beyond the first year after delivery, even after 2 years [11]. Compared with mothers who did not see a psychiatric doctor in a hospital and/or use antidepressants previously, women who went to see a psychiatric doctor in a hospital and/or use antidepressants after first birth have a higher risk of recurrence of postpartum depression after the second child. [12]. Recurrent major depression with history of PPD in Han Chinese women is typically chronic and severe [13]. Therefore, it is important to identify the potential protective or risk factors for postpartum depression in Chinese women.

Although the association between perceived social support and postpartum depression has been investigated in previous studies, social support and depressive symptoms were simultaneously examined in the postpartum periods in many studies [1, 4, 14, 15]. A few studies have used prospective cohort design [16–18]. Three prospective studies in Asia investigated the association between social support during pregnancy and postpartum depression. One study in Japan (*N* = 877) suggested that satisfaction with social support during pregnancy was not a significant factor of maternal postpartum depression [16]. One study in China (*N* = 534) showed that low levels of subjective support were relevant to PPD [17]. The other study in China (*N* = 240) suggested that low perceived social support received during pregnancy did not predict the risk of postpartum depression [18]. Numerous factors are known to be associated with postpartum depression, such as sleep quality and health status [19–21]. However, no previous studies have investigated these factors.

The effect of perceived social support during pregnancy on postpartum depressive symptoms remains controversial. Therefore, we performed a large prospective study (*N* = 2546) to evaluate the association between perceived social support during pregnancy and postpartum depressive symptoms in a Chinese population.

## Methods

### Subjects and procedures

The Shanghai Birth Cohort recruited women, from 2013 to 2016, who visited antenatal clinics based in Shanghai, China [22]. Our research staff recruited women who came for booking for prenatal care 6 maternity hospitals. They were informed of the purpose of this project and the procedures involved. The 6 maternity hospitals where we recruited participants were located in 4 clusters. Two clusters are in the urban setting, one cluster in semi-rural area, and one cluster in suburban area. The inclusion criteria are as follows: women who were at least 20 years old; Registered residents of Shanghai with no plan to move out of Shanghai for the next two years; the period of pregnancy was less than 16 weeks; had planned to deliver in the recruiting hospitals. Exclusion criteria included history of bipolar disorder, depression, schizophrenia or other psychotic illnesses. During the time of the face-to-face interview, a trained interviewer filled in a standardized questionnaire by requiring the participants to answer the questions regarding demographic characteristics, health-related behaviors, psychosocial health, reproductive and medical history. The data regarding demographic characteristics, health-related behaviors, reproductive and medical history was collected before the 16th of week of gestation. The depression symptoms were assessed at 6 weeks postpartum. Written informed consent was obtained from each participant prior to their participation. The study was approved by the local ethical committee. Data from both the prenatal and postpartum components are used in this analysis. Out of the 3310 expectant mothers during early pregnancy recruited into the Shanghai Birth Cohort Study, 3299 women were eligible after excluded history of bipolar disorder, depression, schizophrenia or other psychotic illnesses.

As shown in Table 1, we excluded 210 subjects out of 3299 women, who have incomplete answers on ESSI and 543 subjects on EPDS. 2546 women were included for investigating the association between perceived social support during pregnancy and depressive symptoms at 6 weeks postpartum in the final analysis. Compared with the total 3299 women, 2546 women were slightly younger on average (28.8 vs. 29.0 years old, *p* = 0.04) (Table 1).

**Table 1:** Comparisons of basic characteristics of included participants and total participants

	Included (*n* = 2546)	Total (*n* = 3299)	*P* *
Mean ± SD			
Age (years)	28.8 ± 3.8	29.0 ± 3.7	0.04
BMI (kg/m^2^) ^a^	21.4 ± 3.0	21.5 ± 3.6	0.53
N (%)			
Ethnicity			0.57
Han	2515 (99.0)	3243 (98.8)	
Others	26 (1.0)	40 (1.2)	
Economic status			0.94
Difficult/careful/loan	306 (12.3)	373 (12.2)	
Fair	1747 (70.0)	2125 (69.6)	
Good	444 (17.8)	554 (18.2)	
Education (years)			0.66
≤ 12	258 (10.2)	314 (9.6)	
12–15	1970 (77.5)	2546 (77.6)	
> 15	313 (12.3)	422 (12.9)	
Parity ^b^			0.40
Nulliparous	2138 (84.0)	2760 (83.7)	
Parous	408 (16.0)	539 (16.3)	
Health status			0.83
Fair/poor/very poor	578 (22.8)	729 (23.4)	
Good	1530 (60.3)	1869 (60.0)	
Very good	429 (16.9)	515 (16.5)	
Sleep quality			0.68
Fair/poor/very poor	1292 (50.8)	1602 (51.4)	
Good/very good	1250 (49.2)	1514 (48.6)	
Smoking			1
No	2530 (99.7)	3255 (99.7)	
Yes	8 (0.3)	10 (0.3)	
Alcohol use			1
No	523 (90.8)	2311 (90.9)	
Yes	53 (9.2)	231 (9.1)	

* t-test for continuous variables, chi-square test for categorical variables

a BMI: body mass index calculated by height and weight

b: except parity variable are complete, missing data of others does not count in cells

### Perceived social support assessment

Perceived social support was assessed before the 16th of week of gestation with the ENRICHD Social Support Instrument (ESSI). The full-length ESSI is a 7-item self-report survey [23], which measured the four defining attributes of social support: the emotional, instrumental, informational, and appraisal (Please see Additional file 1 for the details of the ESSI). For this study, we examined perceived social support and thus omitted marital status and instrumental support from the overall ESSI assessment (items 4 and 7). We used the 5-item scale in which the items were about general feelings of being loved and valued rather than instrumental support. After excluding items 4 and 7 as indicated by the ENRICHD protocol (Version 7.0: https://biolincc.nhlbi.nih.gov/studies/enrichd/), the response options of the remaining 5 items (1, 2, 3, 5, and 6) ranged from 1 (none of the time) to 5 (all of the time). The scores of the 5 items were summed up as a total score ranging from 5 to 25, with higher scores indicating greater perception of support. This 5-item scale was highly correlated with the 7-item version [24]. Dichotomous scoring was created to determine the degree of social support (low or high). Using the criteria of the ENRICHD protocol (Version 7.0), low perceived social support was defined as a score ≤ 3 in terms of at least 2 items and a total score of ≤18. In this study, internal consistency (Cronbach’s alpha) of the Chinese version of the 5-item ESSI was 0.803.

### Assessment of depression symptoms at 6 weeks postpartum

The Edinburg Postnatal Depression Scale (EPDS) [25] was used to assess postpartum depressive symptoms at 6 weeks after childbirth, which contained a total of 10 items. The scale assessed the woman’s mood during the past week. Each item was scored on a four-point Likert scale (0–3). A total score ranging from 0 to 30 points was calculated by summing across items. The Chinese version of EPDS showed satisfactory psychometric properties. Dichotomous scoring was created to determine postpartum depression. The cut-off point was defined as more than 10 to screen postpartum depressive symptoms among Chinese women with a specificity of 86% and a sensitivity of 82% [3]. Studies show that the Chinese version of EPDS had good validity [3].

### Selection of confounders

Factors that were reported to be associated with perceived social support or postpartum depression in previous research and factors adjusted for in previous studies were considered as potential confounder candidates [4, 6]. This includes sociodemographic variables such as age at pregnancy (≤25, 26–30, 31–35, and 36+ years), women’s education (≤ 12, 12–15, 16+ years), and self-assessment of economic status (difficult/careful/loan, fair, good). Pregnancy-related characteristics and health indicators included were: parity (nulliparous, parous), pre-pregnancy body mass index (categories: < 18.5, 18.5–23.9, 24.0–27.9, ≥28) calculated by height and weight, self-assessment of the past month’s health status before 16 weeks’ gestation (fair/poor/very poor, good, very good), self-assessment of the past month’s sleep quality before 16 weeks’ gestation (fair/poor/very poor, good/very good), smoking status (no, yes) and alcohol use (no, yes). A factor was considered as a confounder if it was associated with perceived social support and postpartum depression (using Chi-square analysis) and if it changed the estimate of the association by ≥10% in the logistic regression model I. We adjusted all potential confounder candidates in the logistic regression model II.

### Statistical analysis

Continuous variables were expressed as mean ± standard deviation, and categorical variables as percentage. The t-test and the chi-square test were used to compare continuous and categorical differences between the included and total population, respectively. Chi-square test or Fisher’s exact test was also performed among the women (*n* = 2546) who completed both the ESSI and the EPDS to describe sample characteristics of potential covariates and perceived social support.

At least one variable was missing in 103 of the 2546 women. To address missing covariate data, under assumed mechanism of missing at random, multiple imputation (MI) was conducted via R (Version R 3.3.1 GUI 1.68 Mavericks build) which allowed incorporation of all participants into multivariable models. As opposed to single imputation, it allows for the statistical uncertainty in the imputation [26]. We used MI based on the chained equations approach (MICE) to handle the missing data. All potential confounder variables shown above used in the multivariable model in addition to other variables that provided information for the imputation were contained in the multiple imputation models (eg. the education level of husband).

Multivariable logistic regression was performed after MI to assess the effect of social support as a categorical variable (low or high) and each item of ESSI as a categorical variable (None/A little/Some, most, all) on postpartum symptoms with adjustment for confounders (model I) and all variables (model II) (Table 3). When each item of ESSI was analyzed as categorical variable, we further conducted a trend test. Logistic regression modeling was utilized to calculate odds ratios (ORs) and 95% confidence intervals (CIs).

We used stratified analyses to evaluate effect modification by confounders. Logistic regression models were performed to analyze the association between social support and postpartum depressive symptoms in strata which were defined by confounders. For the investigation of the modification of social support effects by confounders, stratum × social support interaction terms was introduced into the model.

All statistical analyses were performed with the R Studio (Version 1.0.136) and R (Version R 3.3.1 GUI 1.68 Mavericks build) for mac. The reliability was performed using Statistical Package for Social Sciences (SPSS, version 23.0) for Mac. All analyses were set at *p* < 0.05 for a two-tailed test of statistical significance.

## Results

### Association of social support during early pregnancy with depressive symptoms at 6 weeks postpartum

Among the total 2546 women, 287 (11.4%) had postpartum depressive symptoms. Approximately two-thirds of women’s age ranged from 20 to 30 years old. According to the classification standard of BMI of China [27, 28], 424 (16.7%) women were overweight and obese. The majority (89.8%) were well educated, who had attended college. 2138 (84.0%) women were primiparous. A higher proportion of women with postpartum depressive symptoms were from a population who felt bad about the economic status, the health level or sleep quality (Table 2). Women with depressive symptoms at 6 weeks postpartum were more likely to receive lower social support and to have alcohol-use in addition to having lower economic status, health level or sleep quality according to self-assessment.

**Table 2:** Characteristics of women by EPDS, *n* = 2555

	Total	EPDS		*P* *
	*n* (%)	< 10 (*n* = 2259)	≥10 (*n* = 287)	
		n (%)	n (%)	
Age (years) ^a^				0.50
< =25	471 (20.8)	421 (89.4)	50 (10.6)	
26–30	1314 (59.2)	1161 (88.4)	153 (11.6)	
31–35	594 (26.3)	522 (87.9)	72 (12.1)	
> =36	150 (6.6)	138 (92)	12 (8)	
BMI (kg/m^2^) ^b^				0.65
< 18.5	379 (15.0)	330 (87.1)	49 (12.9)	
18.5–23.9	1726 (68.3)	1534 (88.9)	192 (11.1)	
24.0–27.9	333 (13.1)	298 (89.5)	35 (10.5)	
≥ 28	91 (3.6)	83 (91.2)	8 (8.8)	
Ethnicity ^c^				0.53
Han	2520 (99.0)	2237 (88.8)	283 (11.2)	
Others	26 (1.0)	22 (84.6)	4 (15.4)	
Economic status ^d, j^				< 0.001
Difficult/careful/loan	306 (12.3)	248 (81.0)	58 (19.0)	
Fair	1747 (70.0)	1561 (89.4)	186 (10.6)	
Good	444 (17.7)	411 (92.6)	33 (7.4)	
Education (years) ^e^				0.40
≤ 12	258 (10.2)	232 (89.9)	26 (10.1)	
12–15	1970 (77.5)	1753 (89.0)	217 (11.0)	
> 15	313 (12.3)	271 (86.6)	42 (13.4)	
Parity				0.048
Nulliparous	2138 (84.0)	1886 (88.2)	252 (11.8)	
Parous	408 (16.0)	373 (91.4)	35 (8.6)	
Health status ^f, j^				< 0.001
Fair/poor/very poor	578 (22.8)	467 (80.8)	111 (19.2)	
Good	1530 (60.3)	1382 (90.3)	148 (9.7)	
Very good	429 (16.9)	401 (93.5)	28 (6.5)	
Sleep quality ^g, j^				< 0.001
Fair/poor/very poor	1292 (50.8)	1093 (84.6)	199 (15.4)	
Good/very good	1250 (49.2)	1162 (93.0)	88 (7.0)	
Smoking ^h^				0.23
No	2530 (99.7)	2247 (88.8)	283 (11.2)	
Yes	8 (0.3)	6 (75)	2 (25)	
Alcohol use ^i^				0.003
No	2311 (90.9)	2066 (89.4)	245 (10.6)	
Yes	231 (9.1)	189 (81.8)	42 (18.2)	
Social support				< 0.001
Low	279 (11.0)	223 (79.9)	56 (20.1)	
High	2267 (89.0)	2036 (89.8)	231 (10.2)	

BMI: body mass index calculated by height and weight

^a^ Missing data on 17 women; ^b^ Missing data on 17 women; ^c^ Missing data on 5 women; ^d^ Missing data on 49 women; ^e^ Missing data on 5 women; ^f^ Missing data on 9 women; ^g^ Missing data on 13 women; ^h^ Missing data on 8 women; ^i^ Missing data on 4 women

^j^ Economic status, Health status, Sleep quality were self-assessed by participants

^*^ Calculated by chi-square test except for ethnicity and Smoking which were calculated by Fisher’s exact test

The analysis of multivariable logistic regression after MI showed that lower social support during early pregnancy was associated with 62% increased risk of developing postpartum depressive symptoms for model I (OR: 1.63; 95% CI: 1.15–2.30) and 77% increased risk of developing postpartum depressive symptoms for model II (OR: 1.77; 95% CI: 1.24–2.52). At the same time, for each sub-item analysis, we found that low perceived social support in different aspects was associated with increased risk of PPD and that the less women perceive support in different aspects, the higher the risk of postpartum depression (*p* for trend < 0.001) (Table 3). As shown in Table 4, there was little evidence of the modification effect of social support by confounders.

**Table 3:** Association between total score and individual item scores of perceived social support during early pregnancy and postpartum depressive symptoms

	PPD/Non-PPD	Crude OR (95%CI)	Adjusted OR (95%CI)	
			Model I†	Mode II‡
Perceived social support				
low	56/223	2.21 (1.59–3.08)	1.63 (1.15–2.30)	1.77 (1.24–2.52)
high	231/2036	Reference	Reference	Reference
Item 1				
None/A little/Some	74/353	2.20 (1.61–3.00)	1.62 (1.16–2.26)	1.73 (1.24–2.42)
Most	87/584	1.56 (1.17–2.09)	1.40 (1.04–1.90)	1.36 (1.00–1.85)
All	126/1322	Reference	Reference	Reference
*p* for trend		< 0.001		
Item 2				
None/A little/Some	81/436	2.09 (1.54–2.85)	1.58 (1.14–2.20)	1.59 (1.14–2.22)
Most	99/617	1.81 (1.35–2.42)	1.65 (1.23–2.24)	1.60 (1.18–2.16)
All	107/1206	Reference	Reference	Reference
*p* for trend		< 0.001		
Item 3				
None/A little/Some	26/88	2.62 (1.64–4.18)	1.89 (1.16–3.07)	2.00 (1.22–3.29)
Most	52/321	1.43 (1.03–2.00)	1.32 (0.94–1.85)	1.22 (0.87–1.73)
All	209/1850	Reference	Reference	Reference
*p* for trend		< 0.001		
Item 5				
None/A little/Some	48/179	2.51 (1.76–3.58)	1.85 (1.27–2.70)	2.05 (1.40–3.01)
Most	63/431	1.37 (1.01–1.86)	1.20 (0.87–1.65)	1.17 (0.85–1.62)
All	176/1649	Reference	Reference	Reference
*p* for trend		< 0.001		
Item 6				
None/A little/Some	76/363	2.05 (1.51–2.77)	1.57 (1.14–2.16)	1.64 (1.19–2.28)
Most	72/537	1.31 (0.97–1.77)	1.19 (0.87–1.63)	1.17 (0.85–1.60)
All	139/1359	Reference	Reference	Reference
*p* for trend		< 0.001		

† model I Perceived social support, Item 2, 5, 6: adjustment for health state, sleep quality, economic status; Item 1: adjustment for health state, BMI, sleep quality, economic status; Item 3: adjustment for health state, sleep quality

**‡** model II: adjustment for age, education, BMI, health state, sleep quality, economic status, smoking state, alcohol use, parity, ethnicity

**Table 4:** Association between social support and postpartum depressive symptoms in strata defined by the potential confounder candidates

	*n* (%)	OR (95% CI) *	*P* for effect modification
Economic status			0.83
Difficult/careful/loan	355 (13.4)	1.89 (1.00–3.57)	
Fair	1796 (67.9)	1.44 (0.92–2.28)	
Good	493 (18.6)	1.28 (0.36–4.57)	
Health level			0.59
Fair/poor/very poor	587 (22.9)	1.33 (0.81–2.18)	
Good	1539 (60.0)	1.82 (1.07–3.09)	
Very good	438 (17.1)	1.95 (0.53–7.07)	
Sleep quality			0.51
Fair/poor/very poor	1292 (50.8)	1.49 (0.99–2.26)	
Good/very good	1250 (49.2)	1.74 (0.90–3.37)	

Inf: infinity

* Calculated by logistic regression for the association between social support and postpartum depressive symptoms

## Discussion

We have performed the largest prospective study in a Chinese population to investigate the association between perceived social support during pregnancy and postpartum depressive symptoms. We found that lower perceived social support in pregnancy was associated with higher risk of depressive symptoms at 6 weeks postpartum.

The effect of perceived social support during pregnancy on PPD has been examined by the previous studies, but only a few conducted cohort studies. However, most of them did not measure the depressive state at the time of evaluation or investigate sleep quality [19, 21] and health status that are associated with postpartum depression. Poor sleepers may feel more pessimistic [29] and thereby underestimate their own social support status. Therefore, it is essential to evaluate perceived social support during pregnancy with taking sleep quality into account at the same time.

In our study, the results show inverse associations between perceived social support and postpartum depressive symptoms, while two previously cohort studies suggested that they were not significantly associated. Morikawa et al. [16] evaluated the association between pregnant women’s satisfaction with social support by using satisfaction subscales of the Japanese version of the abbreviated Social Support Questionnaire (J-SSQ). The results demonstrated that satisfaction with social support during pregnancy was not significantly associated with the postpartum depression identified by EPDS. However, they did not investigate some factors that are known to be associated with postpartum depression, such as sleep quality. In the present study, logistic regression analysis was performed treating them as confounders. Li et al. [18] obtained similar results but again did not investigate some factors that are known to be associated with postpartum depression. Our results are similar to the earlier prospective study where they found inverse associations between perceived social support and postpartum depressive symptoms [30]. However, most of them did not investigate sleep quality and health status that are associated with postpartum depression. Variation in assessments and time points of social support and postpartum depression makes it difficult to compare results across studies.

There are several strengths in this study. First, this is the largest prospective study that has investigated the association between social support and postpartum depression symptoms. Second, we investigated sleep quality and health status that are associated with postpartum depression. People with poor sleep may feel depressed and think they are receiving low social support. Third, women with previous psychiatric illness were excluded, which were assessed by asking if women had ever been diagnosed with bipolar disorder, depression, schizophrenia or other psychotic illnesses. Finally, depression is a chronic disease and long-term exposure may have a large impact on it. Therefore, studying exposure at early stage may be important for preventing postpartum depression. Several limitations of our study should be considered. First, we have assessed women’s history of depression, but have not measured the depressive state at the time of evaluation. Depressed individuals are more pessimistic and tend to underestimate the social support they perceive. Furthermore, depression is closely related to postpartum depression. The recent state of depression should be included as a covariate in the future research models. Second, sleep quality and health status have been assessed with self-assessment questions which were not a standard and valid questionnaire for data analysis. Last but not least, we assessed postpartum depressive symptoms using the EPDS which is a method of identifying possible postpartum depression but not a tool for ascertaining the diagnosis.

## Conclusion

We observed that women with lower perceived social support during pregnancy may have an increased risk of depressive symptoms after childbirth. Early intervention may be able to prevent depression symptoms at 6 weeks postpartum.

## Additional file

Additional file 1: ENRICHD Social Support Instrument. (DOCX 69 kb)

## Data Availability

The datasets analyzed during the current study are not publicly available but are available from the corresponding author on reasonable request.

## Abbreviations

CI: confidence interval
EPDS: Edinburgh Postpartum Depression Scale
ESSI: ENRICHD Social Support Instrument
J-SSQ: Japanese version of the abbreviated Social Support Questionnaire
MI: multiple imputation
OR: odds ratio
PPD: postpartum depression
