# Supplementary material for: The effect of perceived social support during early pregnancy on depressive symptoms at 6 weeks postpartum: a prospective study
Source: BMC Psychiatry. 2019 Jul 29;19:232. doi: 10.1186/s12888-019-2188-2 (PMC6664519; doi:10.1186/s12888-019-2188-2)
Supplement: Supplementary file 1 — ENRICHD Social Support Instrument. (DOCX 69 kb) [file 12888_2019_2188_MOESM1_ESM.docx]

| **ENRICHD Social Support Instrument** | | | | | | | | | | |
| --- | --- | --- | --- | --- | --- | --- | --- | --- | --- | --- |
| **Item 1** |  | Is there someone available to whom you can count on to listen to you when you need to talk? | | | | | | | | |
|  |  | None of the time | | A little of the time | | Some of the time | | Most of the time | | All of the time |
|  |  | ☐ | | ☐ | | ☐ | | ☐ | | ☐ |
| **Item 2** |  | Is there someone available to you to give you good advice about a problem? | | | | | | | | |
|  |  | None of the time | A little of the time | | Some of the time | | Most of the time | | All of the time | |
|  |  | ☐ | ☐ | | ☐ | | ☐ | | ☐ | |
| **Item 3** |  | Is there someone available to you who shows you love and affection? | | | | | | | | |
|  |  | None of the time | A little of the time | | Some of the time | | Most of the time | | All of the time | |
|  |  | ☐ | ☐ | | ☐ | | ☐ | | ☐ | |
| **Item 4** |  | Is there someone available to help with daily chores? | | | | | | | | |
|  |  | None of the time | A little of the time | | Some of the time | | Most of the time | | All of the time | |
|  |  | ☐ | ☐ | | ☐ | | ☐ | | ☐ | |
| **Item 5** |  | Can you count on anyone to provide you with emotional support (talking over problems or helping you make a difficult decision)? | | | | | | | | |
|  |  | None of the time | A little of the time | | Some of the time | | Most of the time | | All of the time | |
|  |  | ☐ | ☐ | | ☐ | | ☐ | | ☐ | |
| **Item 6** |  | Do you have as much contact as you would like with someone you feel close to, someone in whom you can trust and confide in? | | | | | | | | |
|  |  | None of the time | A little of the time | | Some of the time | | Most of the time | | All of the time | |
|  |  | ☐ | ☐ | | ☐ | | ☐ | | ☐ | |
| **Item 7** |  | are you currently married or living with a partner? ☐Yes ☐ No | | | | | | | | |
